# To the Editors of the Medical and Physical Journal

**Published:** 1801-04

**Authors:** Montgomery Boswell

**Affiliations:** Surgeon, Royal Navy. His Majesty's Ship Gibraltar, cruising off Cadiz


### *To the Editors of the Medical and Phyfical Journal.*

Gentlemen,

IN the as yet undetermined ftate of the public mind, relative to the utility of inoculating with the Cow-pox, I am induced to lay before you the following cafe, which happened on board his Majefty's fhip Gibraltar, in Auguft laft. If you fhould deem it worthy of a place in your moft valuable Publication, you are at liberty to infert it. I am, &c.

MONTGOMERY BOSWELL,
Surgeon, Royal Navy.

*His Majesty's Ship Gibraltar, cruising off Cadiz, Jan.* 12, 1801.

---

William Burnell, landfman, aged about 42 years, when fervant to a farmer in Devonfhire, had the Cow-pox; fince which period he has entered into his Majefty's navy. In Auguft laft the variolous infection having made its appearance amongft the fhip's company, thirteen of whom having caught it, did well, it proving to be a very fine pock of the diftinct kind. This man, viz. William Burnell, being one of the people appointed to attend the fick, (and never having had the Small-pox) was anxious that I fhould inoculate him, which I did; and to fecure fuccefs, inferted the variolous matter in both arms. On the fourth morning I found a confiderable degree of inflammation, which appeared to indicate a certainty of his having the Small-pox; but in fix days after, the inflammation totally fubfided, without any puftule having formed. At the expiration of three weeks, I again inoculated him in the manner as mentioned above, producing exactly the fame appearance as before.

---

